# Use of Cellular-Enabled Glucometer for Diabetes Management in High-Risk Pregnancy

**DOI:** 10.1089/tmr.2023.0033

**Published:** 2023-10-12

**Authors:** Rebecca D. Jones, Cheng Peng, Lettie Odom, Heather Moody, Hari Eswaran

**Affiliations:** University of Arkansas for Medical Sciences, Little Rock, Arkansas, USA.

**Keywords:** cellular-enabled remote patient monitoring device, diabetes management, pregnancy, remote patient monitoring, type 1 diabetes, type 2 diabetes

## Abstract

**Background:**

Type 1 and type 2 diabetes during pregnancy requires intensive glucose monitoring to ensure optimal health outcomes for mothers and infants. Standard practice includes patients monitoring their glucose four to six times a day using a standard glucometer and paper diary. Remote patient monitoring (RPM) offers an alternative method for diabetes management. This study aimed at measuring the patient's satisfaction with and feasibility of using a cellular-enabled RPM device for glucose management in pregnancies complicated by type 1 or type 2 diabetes.

**Methods:**

In a mixed-methods pilot study, 59 pregnant women with type 1 or type 2 diabetes were given a cellular-enabled iGlucose glucometer. Participants completed a pre-survey, used the device for 30 days, and then completed a post-survey and semi-structured interview.

**Results:**

Participants were divided into two groups based on duration of device use: high-use >50 days and low-use ≤50 days. A significant difference (*p* < 0.0001) in Appraisal of Diabetes scores was seen between the pre- and post-survey for both groups, which indicates that the use of iGlucose glucometer significantly improved participants' appraisal of their diabetes. There was a significant difference (*p* = 0.0409) in pre-post General Life Satisfaction in the high-use group, which indicates that iGlucose glucometer significantly improved participants' life satisfaction when used for an extended amount of time. Participants scored high on system usability for all groups and reported positive associations with iGlucose use.

**Conclusion:**

The use of cellular-enabled RPM glucometers is a valuable tool for the management of type 1 diabetes mellitus and type 2 diabetes mellitus during pregnancy.

## Introduction

Type 1 diabetes mellitus (T1DM) and type 2 diabetes mellitus (T2DM) during pregnancy affects one out of six live births worldwide and requires intensive glucose monitoring to ensure optimal health outcomes for mothers and infants.^1^ Complications due to glycemic variability during pregnancy include hypertension, C-section, preterm birth, stillbirth, cholestasis, neonatal hypoglycemia, macrosomia, and birth defects.^2–5^ Women with T1DM and T2DM have poorer pregnancy outcomes than women without diabetes, including a higher rate of perinatal mortality.^2^

Hemoglobin A1C (A1C) is used as a measure of glycemic stability and provides an “average” of the patient's blood glucose over the past to 2–3 months.^6^ Optimizing glycemic stability during the first and second trimester can help prevent complications.^2^ Management of diabetes during pregnancy is challenging for both patients and medical providers.

Standard practice includes a patient monitoring their glucose four to six times a day using a standard glucometer and paper diary, discussed with the provider at weekly clinic appointments. Keeping an accurate diary is a challenge for many patients, and weekly clinic visits place a disproportionate burden on patients living in rural areas.

Remote patient monitoring (RPM), such as the iGlucose RPM (Smart Meter, Tampa, Florida) glucometer (hereafter, “iGlucose”), is an alternative method to provide accurate glucose readings while minimizing clinic visits for those living in rural, low-health resource settings. iGlucose is cellular-enabled and automatically transmits glucose readings to a physician portal, reducing the burden for patients by eliminating paper diaries and minimizing clinic visits.

It also ensures health care providers receive accurate glucose readings to manage treatment plans, which is imperative to decreasing the rates of complications seen in pregnancies complicated by DM. To our knowledge, this is the first study to assess the use of cellular-enabled glucometers for the management of DM during pregnancy.

### Purpose

This study aimed at measuring feasibility, clinical impact, and patients' satisfaction with using iGlucose for glucose management in pregnancies complicated by T1DM or T2DM.

## Methods

### Study design

This study was a mixed-methods pilot study utilizing a pre-post survey design and semi-structured qualitative interview to assess the feasibility and acceptability of using iGlucose to manage diabetes during pregnancy. Fifty-nine pregnant women with T1DM or T2DM were invited to participate in the study by a trained research associate. The mean gestational age of participants at the time of consent was 19.73 weeks (standard deviation [SD]: 7.67, range: 28.5).

Participants were provided with iGlucose for home use in accordance with their health care provider's management plan. Because iGlucose is equipped with cellular transmission capabilities, patients do not need Wi-Fi or Bluetooth to operate the device, eliminating the need for a smart phone and creating a greater level of ease and access for those in underserved or rural areas.

Participants were asked to complete a baseline survey, use iGlucose for 1 month, and then complete an exit survey and semi-structured interview. Participants were able to continue using the device after the study, if desired.

### Participants

Participants were women whose pregnancy was complicated by T1DM or T2DM. Inclusion criteria included women (1) 18 years or older who received prenatal care at the University of Arkansas for Medical Sciences (UAMS) Women's Health Clinic, (2) had a pregnancy complicated by T1DM or T2DM, and who (3) spoke English. Exclusion criteria included (1) women who are unable to use a glucometer by themselves.

Fifty-nine women who met the eligibility criteria consented to participate; however, 9 women were lost to follow-up, and 1 participant was excluded due to missing data. Participants received a $15 gift card as compensation following the completion of each survey and a $20 gift card following the completion of the exit interview. The study was approved by the UAMS Institutional Review Board on December 7, 2020 (No. 261693).

### Data collection

#### Quantitative data

A trained research assistant obtained informed consent verbally. Participants completed a web-based, self-administered survey at baseline, consisting of 10 sections: (1) demographic characteristics, (2) treatment engagement, (3) appraisal of diabetes, (4) general life satisfaction, (5) general self-efficacy, (6) depression, (7) anxiety, (8) perceived stress, (9) technology anxiety, and (10) technology facilitating factors. After using iGlucose for a month, participants were asked to complete a post-survey consisting of 11 sections: (1) maternal health outcomes, (2) treatment engagement, (3) appraisal of diabetes, (4) general life satisfaction, (5) general self-efficacy, (6) depression, (7) anxiety, (8) perceived stress, (9) technology anxiety, (10) technology facilitating factors, and (11) system usability. Surveys took ∼15–20 min to complete.

All survey instruments used in this study, except for the treatment engagement, technology anxiety, and technology facilitating factors instruments, were developed and validated for research purposes. The latter three instruments were custom developed by the Antenatal and Neonatal Guidelines Education and Learning System (ANGELS) at UAMS.

The ANGELS research group developed these three instruments because a short and validated survey that effectively assessed participants' treatment engagement levels, levels of anxiety toward technology, and the availability of resources, knowledge, and support at home to engage with the technology could not be identified. We provide both pre- and post-Cronbach's alpha for these instruments to ensure transparency in our study.

#### Qualitative data

Participants were interviewed over the phone by a trained research associate using a semi-structured interview guide at the conclusion of the study. The interview consisted of six questions, including “What were your feelings about using the mobile health monitoring device?,” “Do you think the iGlucose monitoring had a positive or negative effect on your anxiety?,” “Do you think the iGlucose monitoring had a positive or negative effect on your stress level?,” “Do you think your care was better, worse or the same as other women who do not have iGlucose monitoring?,” “What were the advantages of using the iGlucose mobile health monitoring device?,” and “What were the disadvantages of using the iGlucose mobile health monitoring device?” Interviews lasted on average 5–10 min. All interviews were audio-recorded and transcribed verbatim.

### Measurements

#### Treatment engagement

Treatment engagement was assessed using three 7-point Likert scale questions, including “How often did you forget to monitor yourself?,” “How frequently do you feel you were careless about monitoring yourself?,” and “When you felt better, how often did you stop monitoring yourself?.” Responses ranged from 1 (*Never*) to 7 (*Always*), with lower composite scores correlated to higher levels of treatment engagement. The Cronbach's alpha for the pre-survey was 0.75 and for the post-survey, it was 0.82.

#### Appraisal of diabetes

Appraisal of diabetes was assessed using a validated self-report instrument, consisting of seven items. A composite measure of appraisal of diabetes was created by summing individual scores with a range of 7 to 35.^7^

#### General Life Satisfaction

The Patient-Reported Outcomes Measurement Information System (PROMIS) General Life Satisfaction Short Form, a five-item validated self-report instrument, utilizes a seven-point Likert scale to measure general life satisfaction. Higher composite scores indicate higher general life satisfaction.^8^

#### General Self-Efficacy

The PROMIS General Self-Efficacy Short Form, a four-item validated self-report instrument, utilizes a five-point Likert scale to measure general self-efficacy. Higher composite scores indicate higher general self-efficacy.^9^

#### Depression

Depression was assessed using the PROMIS Depression short form consisting of four 5-point Likert scale questions; higher composite scores correlate with higher severity of depression.^10^

#### Anxiety

The PROMIS Anxiety Short Form, a four-item validated self-report instrument, uses a five-point Likert scale to assess anxiety; higher composite scores correlate with higher severity of depression.^11^

#### Perceived Stress Scale

Perceived stress was assessed using Cohen's Short Form Perceived Stress Scale (PSS-4), a self-report instrument consisting of four questions. Questions are rated on a five-point Likert scale ranging from 0 (*Never*) to 4 (*Very often*).^12^

#### Technology anxiety

Technology anxiety was assessed using four Likert scale questions, with higher sum composite scores correlating with higher technology anxiety. Questions included “Using technology makes me nervous.,” “Using technology makes me uncomfortable,” and “Using technology makes me uneasy.” The fourth question, “Using technology does not scare me at all” was reverse coded. The Cronbach's alpha for the pre-survey was 0.65 and for the post-survey, it was 0.79.

#### Technology facilitating factors

Technology facilitating factors were assessed using three 7-point Likert scale questions measuring the participants' resources, knowledge, and support at home to engage with the technology. The Cronbach's alpha for the pre-survey was 0.87 and for the post-survey, it was 0.80.

#### System usability

To assess satisfaction with the usability of the device, a 10-item validated measure of System Usability was used.^13^ Participants rated their experience of using the device from a variety of aspects of system usability, such as the need for support, training, and complexity. A composite measure of the overall usability of the device was created by summing the score from each item and multiplying by 2.5.^13^ The System Usability composite score has a range of 0 to 100.^13^

#### Maternal health outcomes

Maternal health outcomes were assessed using four criteria: (1) number of emergency room visits for DM complications, (2) number of hospitalizations for DM complications, (3) number of calls to the 24/7 High Risk Pregnancy Program Call Center at UAMS for diabetes concerns, and (4) A1C values. Data were obtained from patient charts in EPIC (Epic Systems Corporation, Verona, WI).

### Data analysis

#### Quantitative data

Hot-Deck imputation^14^ was used to impute missing values in survey responses. Participants were divided into classes based on their demographic characteristics, including marital status, highest education level, and annual household income. Within each class, one response donor, who did not have missing items, was assigned to one participant with missing items using Nearest-Neighbor Hot-Deck. Participant race and employment status were further used to define the distance between observations, and the person who is “closest” to the participant with the missing item was chosen as the donor to provide the imputed value.

This study employed a pre-post research design to evaluate the influence of using the iGlucose on study measures. The paired *t*-test and Wilcoxon signed rank test were used to compare the study measures of participants before and after using iGlucose. Measures were also compared by the duration of iGlucose system use, with groups stratified into high-use and low-use group based on the number of days the device was used.

A participant was assigned to the high-use group if they used the device for >50 days and the low-use group if they used the device for 50 days or less. In addition, the differences between each group's pre- and post-study measures were analyzed using the two-sample *t*-test and Wilcoxon Mann–Whitney *U* Test to determine whether there were statistically significant differences between high-use and low-use groups. Data analysis utilized nonparametric tests, such as the Wilcoxon signed rank test and the Wilcoxon Mann–Whitney *U* Test, when normality assumptions were violated. All analyses were conducted using SAS 9.4 software, and an alpha of 0.05 was used to determine statistical significance.

#### Qualitative data

Major themes were identified using the MAXQDA Plus 20 qualitative analysis software^15^ by two researchers using thematic analysis. First, all interview transcripts were read by the coders to familiarize themselves with the data. The codebook was then developed in an iterative process of discussion and refinement. Coders used constant comparative analysis to search line by line for patterns, codes, and themes. As new codes and themes emerged, the coders reviewed previous interviews to ensure consistency. After all transcripts were coded, the data analysis team identified major themes and exemplary quotations.

## Results

### Characteristics of the sample

Sociodemographic characteristics of the 49 participants are reported in Table 1. Most participants were Black or African American (69.39%), single (59.57%), had attended at least some college or technical school (65.3%), and reported an annual household income below $20,000 (57.14%). The majority of participants reported that this was not their first pregnancy (85.71%) and not their first pregnancy with diabetes (53.06%). No differences in patient characteristics were observed between the high- and low-use groups.

**Table 1.** Patients' Demographic Characteristics by iGlucose System Use Frequency

Characteristic	Total (***N*** = 49), ***n*** (%)	High-use, ***n*** = 26, ***n*** (%)	Low-use, ***n*** = 23, ***n*** (%)	***p*** ^a^
Race				
Black or African American	34 (69.39)	16 (61.54)	18 (78.26)	0.4088
Other	2 (4.08)	1 (3.85)	1 (4.35)	
White	13 (26.53)	9 (34.62)	4 (17.39)	
Marital status				
Divorced, separated, or widowed	4 (8.51)	0 (0.00)	4 (17.39)	0.0825
Married	14 (29.79)	9 (34.62)	6 (26.09)	
Other	1 (2.13)	0 (0.00)	1 (4.35)	
Single	28 (59.57)	17 (65.38)	12 (52.17)	
Highest education				
9th grade to 12th grade	3 (6.12)	2 (7.69)	1 (4.35)	0.0990
High school graduate or GED	14 (28.57)	6 (23.08)	8 (34.78)	
Some college or technical school	18 (36.73)	7 (26.92)	11 (47.83)	
College graduate or higher	14 (28.57)	11 (42.31)	3 (13.04)	
Annual household income				
<$15,000	13 (26.53)	8 (30.77)	5 (21.74)	0.0500
$15,000 to <$20,000	15 (30.61)	5 (19.23)	10 (43.48)	
$20,000 to <$25,000	1 (2.04)	0 (0.00)	1 (4.35)	
$25,000 to <$35,000	5 (10.20)	1 (3.85)	4 (17.39)	
$35,000 to <$50,000	9 (18.37)	6 (23.08)	3 (13.04)	
$50,000 to <$75,000	4 (8.16)	4 (15.38)	0 (0.00)	
≥$75,000	2 (4.08)	2 (7.69)	0 (0.00)	
Employment				
Full-time	23 (46.94)	11 (42.31)	12 (52.17)	0.1147
Part-time	10 (20.41)	6 (23.08)	4 (17.39)	
Unemployed	11 (22.45)	4 (15.38)	7 (30.43)	
Unemployed-disabled	5 (10.20)	5 (19.23)	0 (0.00)	
No. of children aged <18				
0	12 (24.49)	7 (26.92)	5 (21.74)	0.0514
1	19 (38.78)	14 (53.85)	5 (21.74)	
2	9 (18.37)	3 (11.54)	6 (26.09)	
3	4 (8.16)	0 (0.00)	4 (17.39)	
4	2 (4.08)	1 (3.85)	1 (4.35)	
5 or more	3 (6.12)	1 (3.85)	2 (8.70)	
First pregnancy				
No	42 (85.71)	22 (84.62)	20 (86.96)	1.0000
Yes	7 (14.29)	4 (15.38)	3 (13.04)	
Last baby delivered				
Pre-term	23 (48.98)	11 (42.31)	13 (56.52)	0.2698
Term	14 (28.57)	9 (34.62)	5 (21.73)	
Post-term	2 (4.08)	0 (0.00)	2 (8.70)	
Missing	9 (18.37)	6 (23.08)	3 (13.04)	
Last baby birth weight range				
Less than 1 pound	3 (6.12)	1 (3.85)	2 (8.70)	0.5190
1 to 2 pounds	1 (2.04)	1 (3.85)	0 (0.00)	
2 to 3 pounds	1 (2.04)	1 (3.85)	0 (0.00)	
3 to 4 pounds	3 (6.12)	0 (0.00)	3 (13.04)	
5 pounds to <6 pounds	7 (14.29)	4 (15.38)	3 (13.04)	
6 pounds or more	24 (48.98)	13 (50.00)	11 (47.83)	
Missing	10 (20.41)	6 (23.08)	4 (17.39)	
First pregnancy with diabetes				
No	26 (53.06)	12 (46.15)	14 (60.87)	0.5770
Yes	16 (32.65)	10 (38.46)	6 (26.09)	
Missing	7 (14.29)	4 (15.38)	3 (13.04)	

^a^ Groups were compared using chi-square tests for categorical variables.

### Quantitative results

Table 2 shows models used to compare pre-post survey scores for all measures except for system usability, which was only assessed in the post-survey. Table 2 also depicts models used to compare the high-use and low-use groups to examine how the duration of iGlucose use influenced outcomes. *p*-Values and each scale's mean score and SD are reported. There was a significant difference (*p* < 0.0001) for Appraisal of Diabetes (*M* = 16.65, SD = 4.53) for all participants, and the post-survey mean score (*M* = 15.63, SD = 4.60) indicates that use of the iGlucose significantly improved participants' appraisal of their diabetes.

**Table 2.** Models Comparing Pre- and Post-Scales and Scale Change between High- and Low-Use Groups

Measures	Statistics	Total		***p*** ^a^	High-use		***p*** ^a^	Low-use		***p*** ^a^	***p*** ^b^
		Pre-survey	Post-survey		Pre-survey	Post-survey		Pre-survey	Post-survey		
Treatment engagement	Mean	11.45	10.98	0.3647	11.15	11.69	0.5844	11.78	10.17	0.0897	0.0555
	Median	11.00	11.00		11.00	11.50		12.00	10.00		
	SD	4.18	4.54		3.97	4.33		4.48	4.73		
Appraisal of diabetes	Mean	16.65	15.63	<.0001	17.62	16.46	<.0001	15.57	14.70	0.0001	0.7112
	Median	17.00	15.00		17.50	16.00		15.00	15.00		
	SD	4.53	4.60		3.80	4.06		5.11	5.07		
PROMIS General Life Satisfaction	Mean	23.16	24.35	0.0663	23.04	24.92	0.0409	23.30	23.70	0.6678	0.2408
	Median	24.00	25.00		24.50	24.50		24.00	26.00		
	SD	6.91	6.56		6.97	5.20		7.00	7.89		
PROMIS General Self Efficacy	Mean	17.02	16.82	0.7526	16.65	16.00	0.2635	17.43	17.74	0.4058	0.3140
	Median	17.00	17.00		17.00	16.00		18.00	18.00		
	SD	2.65	3.06		2.73	3.37		2.56	2.42		
PROMIS Depression	Mean	6.18	6.06	0.5370	6.08	5.73	0.3665	6.30	6.43	1.0000	0.2642
	Median	5.00	4.00		5.00	4.00		4.00	5.00		
	SD	2.88	3.53		2.64	3.13		3.20	3.98		
PROMIS Anxiety	Mean	7.14	7.14	0.9577	7.46	7.19	0.6294	6.78	7.09	0.5764	0.3733
	Median	6.00	6.00		6.00	6.00		5.00	6.00		
	SD	3.63	3.81		3.77	3.71		3.53	4.01		
Perceived Stress Scale	Mean	5.37	5.22	0.7088	5.31	5.23	0.8918	5.43	5.22	0.6790	0.8560
	Median	5.00	6.00		5.00	5.50		6.00	6.00		
	SD	2.89	3.18		2.66	3.12		3.19	3.32		
Technology anxiety	Mean	7.80	7.22	0.2351	8.54	8.00	0.4374	6.96	6.35	0.3610	0.7415
	Median	8.00	6.00		9.00	8.00		5.00	4.00		
	SD	3.73	4.17		3.49	4.45		3.89	3.74		
Technology facilitating factors	Mean	17.80	18.00	0.8658	16.85	17.96	0.3077	18.87	18.04	0.2480	0.3048
	Median	18.00	19.00		18.00	18.00		18.00	19.00		
	SD	3.98	3.98		5.00	4.18		1.96	3.84		
System usability	Mean	—	81.63	—	—	85.19	—	—	77.61	—	0.4298
	Median	—	90.00		—	90.00		—	77.50		
	SD	—	18.35		—	14.00		—	21.92		

^a^ *p*-Values calculated using paired *t*-test and Wilcoxon signed rank test.

^b^ *p*-Values calculated using the two-sample *t*-test and Wilcoxon Mann–Whitney *U* test.

PROMIS, Patient-Reported Outcomes Measurement Information System; SD, standard deviation.

A statistically significant difference in Appraisal of Diabetes was also observed among both the high-use (*p* < 0.0001) and low-use groups (*p* < 0.0001). There was a significant difference in the high-use group's pre-post PROMIS general life satisfaction scores (*p* = 0.0409), indicating that iGlucose significantly improved participants' life satisfaction when used for an extended amount of time. No differences were observed between pre-post mean scores and high versus low-use groups for any other measures.

Participants scored high on system usability with mean scores of 81.63 (SD = 18.35) for the whole group, 85.19 (SD = 14.00) for the high-use group, and 77.61 (SD = 21.92) for the low-use group, indicating that iGlucose users perceived the technology to be easy to use and beneficial.

Table 3 compares health care utilization and A1C values between the high-use and low-use groups. Since the distribution of these study measures was not normal, the nonparametric Wilcoxon Mann–Whitney *U*-test was used to determine whether there were differences between the two groups. There was no difference observed between the two groups in terms of health care utilization.

**Table 3.** Health Care Utilization and A1C Values Between High- and Low-Use Groups

Measures	Total, ***M*** (SD)	Total, range	High-use, ***M*** (SD)	High-use, range	Low-use, ***M*** (SD)	Low-use, range	***p*** ^a^
No. of emergency department visits	0.22 (0.59)	3.00	0.27 (0.72)	3.00	0.17 (0.39)	1.00	1.0000
No. of hospitalizations	0.24 (0.80)	5.00	0.27 (1.00)	5.00	0.22 (0.52)	2.00	0.6535
No. of calls to the 24/7 High Risk Pregnancy Program Call Center	0.20 (0.50)	2.00	0.27 (0.60)	2.00	0.13 (0.34)	1.00	0.5404
A1C values	7.09 (1.77)	7.30	7.08 (1.63)	5.20	7.10 (1.96)	7.30	0.8890

^a^ *p*-Values calculated using Wilcoxon Mann–Whitney *U* test.

A1C, hemoglobin A1C.

Although the high-use group has a slightly lower mean A1C value of 7.08 (SD = 1.63) compared with the low-use group's mean of 7.10 (SD = 1.96), the *p*-value of 0.8890 indicates there is no difference between the two groups. The Pearson correlation coefficient for A1C and length of device use was 0.047, which was not significant (*p* = 0.7465).

### Qualitative results

#### Advantages

Of the 49 participants, 42 participants felt that the main advantage of using iGlucose was the automatic transmittal of blood glucose readings to a physician portal and 5 participants believed that iGlucose reduced their number of clinic visits. Thirty participants perceived that they received better care due to the constant monitoring. Six participants identified accurate readings as another positive aspect of using the device, stating that they could no longer record inaccurate blood glucose levels into their paper diary. Thirty-one participants felt the device was convenient or easy to use and 9 participants stated that using the device increased their own self-awareness, allowing for better management of their diabetes.

### Disadvantages

Although most (75.7%) participants reported no issues, a few participants reported concerns with the device. Five participants reported inaccurate glucose readings when compared with other monitors, and nine participants had difficulty with their glucose readings not uploading automatically to the website. Example quotes for each theme are presented in Table 4.

**Table 4.** Exemplary Quotes for Qualitative Themes

Topic	Theme	Representative quotes
Advantages	Automatic upload of blood glucose readings	“I didn't feel like overwhelmed ‘cause with the regular [glucometer] I have to document my readings.” “It was pretty positive because I know I don't have to keep up with the piece of paper. I can monitor my glucose and then it'll just automatically send my readings in.” “It was easier to test ‘cause I didn't have to say where are my logs, where's this, where's that. It was a breeze, so I didn't mind doing it as much.”
	Reduced no. of clinic visits	“I don't necessarily have to go to a doctor every week because they can see my numbers and we can do virtual or chat over the phone.” “I didn't have to come to the doctor… I didn't have to go back and forth and get it checked or go somewhere and get it checked. So that helped a whole lot.”
	Perceived better care	“[My care was] better because they have those accurate numbers right there whenever, I don't have to take time to get them together or forget them.” “It was better ‘cause it goes straight to my doctor so let's them monitor my fluctuations, like if I'm eating something or certain times of the day where I'm peaking more for my blood sugars.” “In comparison to my previous meter and in comparison to what I was eating, the iGlucose seemed to be more accurate than my previous monitor.”
	Accurate readings	“I feel like it's more honest. Because, I'm just saying, if you have another machine, you can write down whatever you want. You can't cheat with this one, what it is, is what's on the screen. So, I think it's better.” “Because, you know, I couldn't like, take it and then drink a bunch of water if I needed a lower sugar level. It makes you tell the truth,” “If you eat wrong, it goes straight to your doctor.”
	Increased self-awareness	“For me, I feel like it made me more aware for each meal, the differences with each meal and how it contributed to my glucose numbers… It just made me more aware.” “It helped me to make sure I was eating healthier because I knew when I took my sugar, the doctor could automatically see my numbers and it's not something that I can kind of fake” “I wasn't taking it seriously until I got here and realized this is something I needed to do and this is something that seriously needs to be done so it gave me that motivation to stay on top of it.”
	Convenient/easy to use	“I thought it was a great way to be able to send my blood sugars over without having to do anything. It's foolproof.” “I definitely feel like I will continue to use it.” “It was probably the smoothest [glucometer] I've ever used” “I'd tell anybody that using the device it is the best thing you can ever ask for… it was one of the easiest ones I've had since I've been dealing with diabetes for a long time.”
Disadvantages	Inaccurate glucose readings	“It gave me inaccurate results, results that were higher than what they really were. I even compared it in the doctor's office and compared it utilizing my own device as well and it had me taking more insulin than what I really needed to take.” “I noticed that when I was checking sometimes it wasn't accurate like the other one…. it would give me lower numbers and sometimes higher numbers.”
	Issues with glucose readings being uploaded	“It's hard when you don't live in a place with a lot of service. Sometimes the readings don't send because there's no service.” “If you're not in that area where you have coverage, like where I live at there's trees and a field area, so some of my readings didn't register.” “Sometimes it would send and sometimes it wouldn't send to the provider.”

## Discussion

This pilot study explored the feasibility and acceptability of using cellular-enabled RPM glucometers to monitor glucose levels in pregnancies complicated by T1DM or T2DM. Previous research on RPM use for diabetes management has primarily focused on non-pregnant individuals or on the use of RPM devices requiring a Bluetooth connection.

To the best of our knowledge, the current study is the first to focus on the use of a cellular-enabled RPM glucometer for the management of T1DM and T2DM in pregnancy. We measured participants' satisfaction, treatment engagement, appraisal of diabetes, general life satisfaction, general self-efficacy, depression, anxiety, perceived stress, technology anxiety, and facilitating factors. We also explored the perceived advantages and disadvantages associated with iGlucose in a semi-structured interview.

The Technology Acceptance Model states that patients' use of technology is primarily influenced by perceived usefulness and ease of use of the technology.^16^ Although no difference was seen in Technology Anxiety or Technology Facilitating Factors between high- and low-use groups, participants from both groups scored high on System Usability, indicating that the technology was easily accepted by participants.

In the qualitative results, many participants expressed that iGlucose was convenient and easy to use, and they perceived that they received better care when using iGlucose. Participants who used iGlucose reported higher scores on the Appraisal of Diabetes and General Life Satisfaction measures, indicating that device use had a positive influence on patient perspectives of their diabetes, the effect their diabetes had on their daily life, and their ability to self-manage their diabetes.

Diabetes management, especially during pregnancy, can be extensive and time consuming for many patients. As a result, patients may record the data later and/or may estimate their blood glucose levels. One study of 62 pregnant women with DM found that only 59.3% of women recorded their blood glucose results in their diary at the time of monitoring the blood glucose level, 28.8% recorded their values at the end of the day, and 11.9% at the end of the week.^17^

Patients also report fabricating data in their paper logs. A study of 85 pregnant women comparing patients self-reported paper logs with values from their glucometer's memory found that accuracy of self-reported patient data was low: T1DM patients inaccurately recorded 36.7% of their glucose values, and T2DM patients inaccurately recorded 8.5% of their values.^18^

These results emphasize the importance of RPM to assist in eliminating inaccurate data and ensuring adequate treatment. Our qualitative results reflect these findings, as participants stated that a major advantage of using iGlucose was that it did not allow for the misreporting of blood glucose readings.

A systematic review of the effectiveness of mHealth interventions on non-pregnant patients with diabetes found that when compared with non-mHealth approaches, mHealth interventions of at least 6 months improve A1C by 0.8% for type 2 diabetics and 0.3% for type 1 diabetics.^19^ Another study found an average reduction of two points in A1C after 12 months of diabetes self-management education provided using an electronic tablet.^20^ The short duration of this study combined with the small sample size may explain why we did not see a significant difference in A1C in our study.

Little research has been done on patient satisfaction with using RPM to manage diabetes during pregnancy. One study involved low-risk pregnancy patients without diabetes participating in a program of text-based telemedicine with vital measurements taken via RPM; patients using telemedicine and RPM demonstrated greater engagement and confidence with their care.^21^ Similar results were seen in this study; patients felt their overall care was better and reported an increase in their own self-awareness and engagement with their diabetes management when using the RPM device.

A difference in self-reported Treatment Engagement pre- and post-use of iGlucose was seen in the low-use group with a pre-survey mean value of 11.78 and a post-survey mean value of 10.17. Although this change was not significant (*p* = 0.08), likely due to the small sample size, it does suggest that use of the RPM glucometer did improve treatment engagement in those who used the device short-term. However, research has indicated that for patients who struggle with diabetes management, RPM is not a long-term solution and more personalized interaction and intervention is needed.^22^

In addition, prior research found that women using public insurance such as Medicaid, who have higher baseline A1C, and/or have reduced engagement in their own care have less success with using RPM devices.^23^ Increasing patient activation can lead to lower A1C.^24^ Our results suggest that although treatment engagement increased among those who used the device short-term, they were unable to maintain engagement. Future research should focus on increasing patient activation before RPM device use to encourage sustained usage.

The study does have a few limitations. The study was a pilot; therefore, the duration of the study was short, and the sample size was small. With a longer intervention time and a larger sample size, we may see a significant effect on maternal clinical outcomes and A1C values. In addition, the main study outcomes were self-reported, and social desirability bias may have influenced both quantitative and qualitative results. Nonetheless, our study provides a foundation for future research assessing the use of cellular-enabled RPM glucometers for diabetes management during pregnancy.

## Conclusion

The use of cellular-enabled RPM glucometers is a valuable tool for the management of T1DM and T2DM during pregnancy. Participants who used iGlucose demonstrated increased positive appraisal of diabetes as well as general life satisfaction. In addition, participants scored the device high on system usability and satisfaction, and overall feedback on the device was positive.

Participants cited many benefits, including automatic data upload, reduced number of clinic visits, perceived better care, increased accuracy of results, increased self-awareness, and that the device was convenient and easy to use. Although some disadvantages such as inaccurate readings and difficulty with automatic data upload were reported, the overall positive feedback and positive results of this study indicate that cellular-enabled RPM glucometers should be considered for the use of managing diabetes during pregnancy.

## Abbreviations Used

A1C: hemoglobin A1C
ANGELS: Antenatal and Neonatal Guidelines Education and Learning System
PROMIS: Patient-Reported Outcomes Measurement Information System
PSS-4: Perceived Stress Scale
RPM: remote patient monitoring
T1DM: type 1 diabetes mellitus
T2DM: type 2 diabetes mellitus
UAMS: University of Arkansas for Medical Sciences
